# An Umbrella Review With Meta-Analysis of Chest Computed Tomography for Diagnosis of COVID-19: Considerations for Trauma Patient Management

**DOI:** 10.3389/fmed.2022.900721

**Published:** 2022-07-26

**Authors:** Andrés Gempeler, Dylan P. Griswold, Gail Rosseau, Walter D. Johnson, Neema Kaseje, Angelos Kolias, Peter J. Hutchinson, Andres M. Rubiano

**Affiliations:** ^1^Centro de Investigaciones Clínicas, Fundación Valle del Lili, Cali, Colombia; ^2^NIHR Global Health Research Group on Neurotrauma, University of Cambridge, Cambridge, United Kingdom; ^3^Division of Neurosurgery, Department of Clinical Neurosciences, Addenbrooke’s Hospital, University of Cambridge, Cambridge, United Kingdom; ^4^Department of Neurosurgery, George Washington University School of Medicine and Health Sciences, Washington, DC, United States; ^5^School of Medicine and Public Health, Loma Linda University, Loma Linda, CA, United States; ^6^Surgical Systems Research Group, Kisumu, Kenya; ^7^Neuroscience Institute, INUB-MEDITECH Research Group, El Bosque University, Bogotá, Colombia; ^8^Neurological Surgery Service, Vallesalud Clinic, Cali, Colombia

**Keywords:** umbrella review, evidence based synthesis, COVID-19, global health, trauma surgery, evidence-based practice, chest CT, trauma

## Abstract

**Background:**

RT-PCR testing is the standard for diagnosis of COVID-19, although it has its suboptimal sensitivity. Chest computed tomography (CT) has been proposed as an additional tool with diagnostic value, and several reports from primary and secondary studies that assessed its diagnostic accuracy are already available. To inform recommendations and practice regarding the use of chest CT in the in the trauma setting, we sought to identify, appraise, and summarize the available evidence on the diagnostic accuracy of chest CT for diagnosis of COVID-19, and its application in emergency trauma surgery patients; overcoming limitations of previous reports regarding chest CT accuracy and discussing important considerations regarding its role in this setting.

**Methods:**

We conducted an umbrella review using Living Overview of Evidence platform for COVID-19, which performs regular automated searches in MEDLINE, Embase, Cochrane Central Register of Controlled Trials, and more than 30 other sources. The review was conducted following the JBI methodology for systematic reviews. The Grading of Recommendations, Assessment, Development, and Evaluation approach for grading the certainty of the evidence is reported (registered in International Prospective Register of Systematic Reviews, CRD42020198267).

**Results:**

Thirty studies that fulfilled selection criteria were included; 19 primary studies provided estimates of sensitivity (0.91, 95%CI = [0.88–0.93]) and specificity (0.73, 95%CI = [0.61; 0.82]) of chest CT for COVID-19. No correlation was found between sensitivities and specificities (ρ = 0.22, IC95% [–0.33; 0.66]). Diagnostic odds ratio was estimated at: DOR = 27.5, 95%CI (14.7; 48.5). Evidence for sensitivity estimates was graded as MODERATE, and for specificity estimates it was graded as LOW.

**Conclusion:**

The value of chest CT appears to be that of an additional screening tool that can easily detect PCR false negatives, which are reportedly highly frequent. Upon the absence of PCR testing and impossibility to perform RT-PCR in trauma patients, chest CT can serve as a substitute with increased value and easy implementation.

**Systematic Review Registration:**

[www.crd.york.ac.uk/prospero], identifier [CRD42020198267].

## Key messages

What is already known on the subject?

RT-PCR testing has disadvantages for detecting COVID-19 timely in a trauma surgery setting. Sample processing can be long and requires specific laboratory protocols, which usually delay test results; and sampling can be difficult during basic and advanced trauma life support. Chest CT has been proposed as an additional tool in the diagnosis of COVID-19, and several reports from primary and secondary studies that assessed its diagnostic accuracy have been published.

What are the new findings?

Chest CT is a highly sensitive tool to detect COVID-19. Chest CT specificity was lower than sensitivity. Great variation was present between studies due to differences in design, index test definition and reference standards.

How might these results affect future research or surgical practice?

Chest CT is valuable when PCR testing is absent or obtaining timely results is not possible. Positive findings on chest CT should prompt additional protective measures in aerosolizing procedures for medical staff and isolating measures for the patient.

As emergency trauma patients typically undergo localized or full body CT scanning, imaging of the lungs and its interpretation by a radiologist is not expected to increase costs significantly and can be implemented as a screening tool in that setting.

One important consideration are patients with trauma or polytrauma involving the chest, as lung contusions and hemo- or pneumo-thorax will affect the readability of chest CT for pneumonia.

## Background

During the COVID-19 pandemic, healthcare facilities all over the world are challenged when caring for trauma patients for whom a history of typical symptoms, close contacts and even vaccination status is not available because of agitation, intoxication or unconsciousness due to injury and sedation. Timely detection of cases is crucial to prompt adequate isolation measures and use of personal protective equipment to protect medical staff and other patients. These measures should be applied preventively in every trauma patient with need for emergency surgery, but shortages and sparse resources limit the compliance of these COVID-19 preventive recommendations in low and middle income countries (LMICs); and are thus applied almost exclusively in confirmed cases (1–3). Diagnosis is done with reverse transcriptase polymerase chain reaction (RT-PCR) to identify genetic material of the virus in nasopharyngeal or oropharyngeal swabs.

RT-PCR testing has some disadvantages that become more important in the emergency surgery setting: sample processing can be long and requires specific laboratory protocols, which usually delays test results; and sampling can be difficult during basic and advanced trauma life support. RT-PCR has varying sensitivity among different sampling modes: 97.2% (95%CI: 90.3–99.7%) in sputum; 62.3% (95%CI: 54.5–69.6%) in saliva; and 73.3% (95%CI: 68.1–78.0%) in nasopharyngeal and throat swabs; with a pooled sensitivity estimated at 84.8%, 95% CI = [76.8%; 92.4%]) (4, 5). Of these, nasopharyngeal and throat swabs are the most commonly applied. For both, a considerable rate of false negative results has been reported and is to be expected: an initial false negative RT-PCR results was measured as high as 54% of the time (4, 6). False positives, on the contrary, are very unlikely because specificity has been reported at 98.9%, 95% CI = [97.4%; 99.8%]) with low variability (4). Chest CT has been proposed as an additional tool in the diagnosis of COVID-19, and several reports from primary and secondary studies that assessed its diagnostic accuracy have been published. Chest CT cannot detect asymptomatic carriers, of course, but could detect COVID-19 pneumonias that single RT-PCR can miss (if available).

The above factors make RT-PCR flawed and unpractical for COVID-19 detection in trauma patients, and open the door for a role of chest CT (6). The reported accuracy of chest CT for COVID-19 diagnosis varies substantially and the validity of primary studies is variable, being affected by poor adherence to reporting guidelines and high risk of bias (7). To inform recommendations and practice regarding the use of chest CT in the in the emergency trauma setting with the above factors in mind, we sought to identify, appraise, and summarize the available evidence on the diagnostic accuracy of chest CT for rapid diagnosis of COVID-19, and discuss important considerations for its use.

## Methods

We conducted a broad evidence synthesis (umbrella review) to summarize the diagnostic accuracy of chest CT imaging to detect COVID-19, the respiratory disease caused by the SARS-CoV-2 virus. A protocol of this review following the PRISMA statement was registered in the International Prospective Registry of Systematic Reviews (PROSPERO; CRD42020198267) and published in *JMIR Research Protocols* (8). This review was conducted following the JBI methodology and the Cochrane Handbook for systematic reviews of diagnostic accuracy (9).

### Study Selection

Selection was carried out in Covidence (Melbourne, Australia). Two independent reviewers examined titles and abstracts for eligibility. Full-text review verified fulfillment of selection criteria. All decisions taken during screening were documented and are outlined in this report with a list of excluded studies. Any disagreements that arose between the reviewers were solved by consensus. The results of the search are presented in a Preferred Reporting Items for Systematic Reviews and Meta-analyses (PRISMA) flow diagram (10).

#### Selection Criteria

##### Population of Interest

We considered studies that assessed chest CT imaging for diagnosis of COVID-19 pandemic in trauma patients. Given the likelihood that reports on this specific population were limited, we also included studies of any patients with clinical suspicion of COVID-19 in any procedural and in-hospital setting as the diagnostic accuracy for detection of COVID-19 was considered extrapolatable to most trauma patients.

##### Types of Studies

Systematic reviews of diagnostic test accuracy studies (DTA) and individual DTAs not included in systematic reviews were included. To be considered a DTA, a study had to include patients with a diagnostic equipoise: patients with and without COVID-19, to accurately measure both sensitivity and specificity. Only studies published in English, Spanish or French were considered. We included pre-print studies identified in our search, but no ongoing studies were searched or considered.

##### Index Test

For eligibility, a study had to report positive or negative findings of COVID-19 in chest CT imaging, or report findings on chest CT imaging according to described radiologic scales such as the CO-RADS classification or the consensus by the Radiological Society of North America (11, 12). Imaging analyses other than positive or negative for COVID-19 were not used for metanalysis but were considered to summarize valuable information on radiologic reporting of COVID-19 chest CT imaging.

### Search Strategy

We conducted searches in the L⋅OVE (Living OVerview of Evidence) platform for COVID-19, a system that performs automated regular searches in PubMed, Embase, Cochrane Central Register of Controlled Trials (CENTRAL), and over thirty other sources (13). When compared to manual searches, this platform consistently identifies all the available studies associated with the terms of interest. It allows for a fast (automated) search that is easy to update – a crucial element given the urgent need to answer the research question rapidly and thoroughly.

The platform was consulted on March 15, 2021, using the entry: (1) Diagnostic – Imaging tests – Computed tomography – Population Filter: COVID-19. The full search strategy and terms used to identify papers in L⋅OVE are presented in our registered protocol. Search through cross-referencing was also carried out to identify additional references.

### Assessment of Methodological Quality

Eligible studies were critically appraised by a reviewer and verified by a second reviewer using the QADAS-2 tool for diagnostic test accuracy studies, and the AMSTAR-2 tool for systematic reviews and meta-analyses (9). The results of the critical appraisal are reported narratively and were considered for discussion of results. All included studies underwent data extraction and appraisal. Studies that applied single RT-PCR as a reference standard were consider to have evident risk of bias for estimating diagnostic accuracy due to the likelihood of an initial false negative result in symptomatic patients. If RT-PCR was not repeated or accompanied by adequate follow-up that discards other etiologies, diagnostic accuracy of CT imaging was likely to be biased, particularly in the calculation of specificity. If a single RT-PCR is used as reference standard, false negatives that could be detected by chest CT are wrongfully considered as false positives of chest CT, gravely distorting actual test accuracy. This aspect was rigorously assessed in primary DTA studies and systematic reviews to avoid metanalysis of studies with high risk of bias that directly and considerably affects estimations of diagnostic accuracy.

### Data Extraction

Data were extracted from the included studies by a reviewer and verified by a second reviewer using a data extraction tool developed by the authors. The data extracted included specific details about the study population, the index test and reference standard used, other sources of bias, frequency of true negatives, false positives, true positives and false negatives. Disagreements were solved by consensus.

### Data Synthesis

A narrative-only summary of review findings was planned. Nonetheless, the included reviews were found to either have high risk of bias in its estimates due to acceptance of single-PCR testing as a reference standard for considering studies in metanalysis, or did not include several of the identified primary DTA studies. This indicated the need to conduct a quantitative synthesis as well, as considered and planned in the published review protocol.

### Meta-Analyses

Estimates of pooled sensitivity and specificity as well as other diagnostic summary measures were obtained by use of the bivariate random-effect model methods (14). This approach is the standard for DTA meta-analyses when sources of heterogeneity additional to threshold effect are expected (as is the case with chest CT for COVID-19), and averaged or weighted univariate methods for metanalysis of sensitivity and specificity are discouraged (14, 15). A bivariate method was considered most adequate as it incorporates unexplained variability in the analysis. Variation in specificity or sensitivity measures between studies can be attributed to differences in index tests, reference standards, study populations and settings. When sensitivity and specificity derive from the cut-off of a scale, they have a negative correlation, which is considered with the bivariate method as well (14). With this method, reports with more precise estimates have more weight. A secondary (sensitivity) meta-analyses was performed considering only studies with low risk of bias. Results are presented with pooled estimates of sensitivity, specificity, diagnostic odds ratio, forest plots and summary receiver operator characteristics (SROC) curve. Meta-analyses were conducted in RStudio version 1.3.10 using the “mada” package (16). Likelihood ratios for positive (LR +) and negative (LR-) results were calculated from pooled accuracy estimates.

#### Index Tests and Reference Standards Considered for Meta-Analyses

A definition of positive or negative chest CT had to be provided for adequate extraction of true positives, false negatives, true negatives and false positives. Index tests definitions were considered adequate if standardized or derived from internationally accepted recommendations for chest CT interpretation in COVID-19 patients (11, 12). Some studies based their index test on the dichotomization of these and other proposed scales for chest CT classification of COVID-19-suspected cases. Studies that did not report a specific positive or negative definition of chest CT results were eligible for metanalysis if information for calculation of the same cut-off dichotomization was possible. A reference standard was considered adequate when consisting of: multiple or repeated PCR-testing; or a composite of epidemiological, clinical and PCR according to World Health Organization (WHO) recommendations, which usually included follow-up to determine COVID-19+ or –. Studies with repeated PCR testing applied only to patients with negative results was considered adequate as a false positive result is highly unlikely, and was considered for primary metanalysis.

### Assessing Certainty in the Findings

The Grading of Recommendations, Assessment, Development, and Evaluation (GRADE) approach for grading the certainty of the evidence was followed. Grading the certainty of the evidence was not planned if adaptation from the identified reviews that used the GRADE approach was considered complete and adequate (17, 18). Although the GRADE handbook suggests to assess evidence quality of diagnostic accuracy based on its impact on outcomes (i.e., the aftermath consequences of misdiagnosing patients), the interest on diagnostic accuracy for this review is not related to COVID-19 outcomes, but rather on the impact it could have for hospital personnel and other patients, because of contagion risk (19). Thus, a described alternative for assessing quality of diagnostic tests evidence of the GRADE approach that focuses on diagnostic accuracy estimates was used instead of considering test accuracy as a surrogate of patient outcomes. Given that pre-test probability of COVID-19 in trauma patients is low, the effect of diagnostic accuracy was assessed with pre-test probabilities of 1 and 10%, reflecting the scenario in which the diagnostic test is to be applied. The certainty of the evidence is reported in a summary of findings (SoF) table and was considered for interpretation and discussion of findings.

### Publication Bias

Adequate methods for assessing publication bias in reviews of diagnostic test accuracy studies have not been developed. Funnel plots to assess asymmetry are designed for use in reviews of randomized trials and should not be used with diagnostic accuracy studies. Some available methods to assess publication bias have low power in the presence of heterogeneity, which is expected in diagnostic reviews, and thus interpretation of statistical evidence for publication bias derived from funnel plot is not recommended, as it does not necessarily imply publication bias (20, 21). Coherently, statistical assessment of publication bias was not undertaken. Furthermore, publication bias is unlikely in diagnostic test accuracy as there are no “positive” or “negative” results that could increase or decreased likelihood of publication, respectively, as is the case in review of other designs. Particularly for this review, risk of publication bias is expected to be low due to its unlikelihood in diagnosis topics and because several pre-print studies were identified and considered.

## Results

### Search Results

**FIGURE 1:**
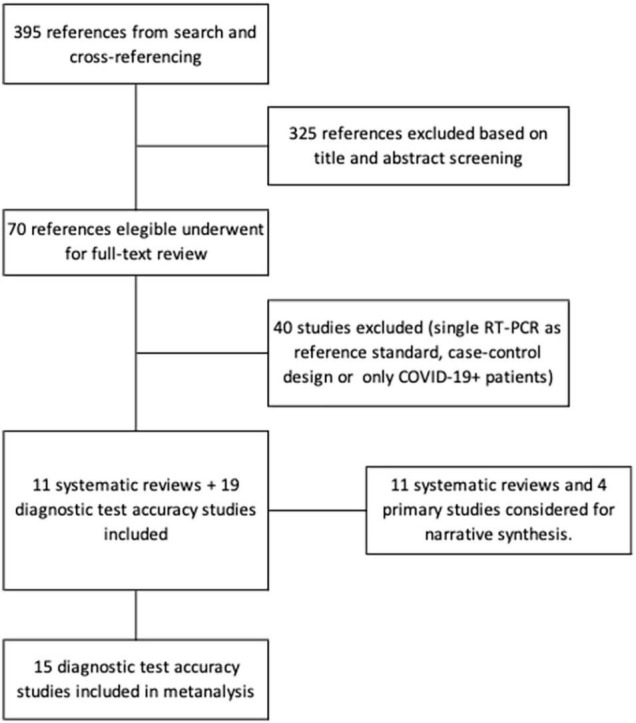
PRISMA flow diagram of the studies selection process.

The search strategy and cross-referencing searches yielded 395 results. After duplicate screening of title and abstract 70 references were selected for full text review of which 30 met selection criteria and were included: 11 systematic reviews (5, 22–31) and 19 primary diagnostic accuracy studies (12, 32–49). Reasons for exclusion were related to not having a DTA design: only COVID-19 patients; only abnormal CT scans; not reporting data to compute sensitivity and specificity; or using single RT-PCR as a reference standard in all patients. Figure 1 is a PRISMA flow diagram depicting the selection process.

None of the identified studies assessed chest CT diagnostic accuracy specifically in trauma patients; all were conducted in the emergency setting in patients with suspected COVID-19. This was expected as low probability of infection in trauma patients makes such studies impractical. As stated in our previously published protocol, the available evidence on chest CT accuracy for diagnosis of COVID-19 was to be considered and summarized, considering the indirectness of the evidence and discussing important considerations for its extrapolation to the trauma setting. Studies conducted in the emergency department setting had only slight differences, but overall, patients underwent chest CT scan examination at admission that was compared to an adequate reference standard for COVID-19; chest CT scan analysis was made by experienced radiologist that were in most cases blinded to reference standard results and determined a positive chest CT scan when patients had an image compatible with or typical of COVID-19. In some studies, no dichotomous (+ or –) index test result was presented, and the results of CT image were described or classified with radiological scales such as the proposed CO-RADS classification or the consensus by the Radiological Society of North America (11, 12). Definition of the index test result as positive or negative was similar and consider comparable among studies. The characteristics of the primary DTA studies considered for synthesis are summarized in Table 1.

**TABLE 1:** Characteristics of studies included in metanalysis.

Citation	Recollection and setting	Selection criteria	Sample size	Reference standard	Chest CT used as Index test reported as ±	Included in meta-analysis
Aslan et al. (32)	Retrospective analysis in the Emergency department with symptomatic patients	At least two of: fever > 38°C, lower respiratory tract infection symptoms suggesting COVID-19, or normal or decreased lymphocyte count and elevated CRP levels; and evaluation by both chest CT imaging and rRT-PCR test at admission. Patients with severe CT motion artifacts or without rRT-PCR testing were excluded.	306	First or repeated rRT-PCR test (repeated if initially negative).	Yes	Yes
Bellini et al. (33)	Retrospective analysis in the Emergency department with symptomatic patients	Patients who underwent chest CT and RT-PCR testing for suspected COVID-19, based on the symptoms: fever higher > 37.5°C, cough, and clinically relevant dyspnea, with or without a history suggestive of exposure to SARS- CoV-2. Exclusion criteria were lack of RT-PCR testing results, time interval between CT scan and RT- PCR longer than 7 days, and uninterpretable CT scans due to motion artifacts or incomplete scanning.	572	Positive RT-PCR or 14-day follow-up with negative diagnosis if no symptoms’ worsening or laboratory findings consistent with COVID-19.	No	No
Caruso et al. (34)	Prospective collection in emergency department with symptomatic patients	Patients with fever and respiratory symptoms as cough and dyspnea; patients with mild respiratory symptoms and close contact with a confirmed COVID-19 patient; patients with a previously positive test result. Exclusion criteria were chest CT with contrast medium performed for vascular indication; patients who refused chest CT or hospitalization; severe motion artifact on chest CT.	158	Two RT-PCR tests with 24 h interval.	Yes	Yes
Debray et al. (35)	Retrospective analysis in the Emergency department with symptomatic patients	Patients presenting with COVID-19 suspicion and for whom hospitalization was considered had both chest CT scan and SARS-CoV-2 RT-PCR.	213	Repeated PCR and clinical features on presentation and follow up. (Although, this standard could not be applied to 28 of the 81 initially negative patients [34.5%], for whom single T-PCR and symptoms were considered)	Yes	Yes
Fujioka et al. (38)	Retrospective analysis in the Emergency department with symptomatic patients	Suspected COVID-19 based on symptoms and history of exposure; who underwent chest CT and were diagnosed as positive or negative for COVID-19 by one or more RT-PCR tests.	154	Diagnosis by an experienced clinician based on chest X-ray, chest CT, laboratory findings, and clinical data in the follow-up and result of RT-PCR.	No	No
Gezer et al. (36)	Retrospective analysis in the Emergency department with symptomatic patients	Adult patients with a chest CT scan upon suspicion of COVID-19 pneumonia with high fever (> 38°C) and respiratory symptoms dyspnea and cough.	222	Diagnosis by consensus of two physicians based on the medical records, CT scans and positive RT-PCR results.	Yes	Yes
Gietema et al. (37)	Prospective collection in emergency department with symptomatic patients	Adult patients with a chest CT scan upon suspicion of COVID-19 pneumonia with high fever (>38°C) and respiratory symptoms dyspnea and cough.	193	Sequential PCR and hospital follow-up, multiple RT-PCR for initially negative.	Yes	Yes
He et al. (48)	Retrospective analysis in the Emergency department with symptomatic patients	Adult patients with a chest CT scan upon suspicion of COVID-19 pneumonia with high fever (>38°C) and respiratory symptoms dyspnea and cough. Patients with incomplete clinical information or excessive motion artifacts on CT were excluded.	82	Multiple RT-PCR testing and clinical observation and follow up.	Yes	Yes
Herpe et al. (39)	Multicenter prospective collection in emergency department with symptomatic patients	Adult patients with a chest CT scan upon suspicion of COVID-19 pneumonia with high fever (> 38°C) and respiratory symptoms dyspnea and cough.	4824	The final discharge diagnosis based on follow-up and COVID-19 criteria.	Yes	Yes
Korevaar et al. (40)	Retrospective analysis in the Emergency department with symptomatic patients	Adult patients with hospital admission that underwent both chest CT and RT-PCR testing for SARS-CoV-2 infection upon admission.	239	COVID-19 criteria and multidisciplinary consensus after follow-up in case of negative RT-PCR testing.	No	No
Krdzalic et al. (41)	Retrospective analysis in the Emergency department with symptomatic patients	Adult patients with a chest CT scan upon suspicion of COVID-19 pneumonia with high fever (>38°C) and respiratory symptoms dyspnea and cough.	56	RT-PCR and sequential retest with RT-PCR in patients with initially negative until persistently negative.	Yes	Yes
Patel et al. (42)	Retrospective analysis in the Emergency department with symptomatic patients	Adult patients with a chest CT scan upon suspicion of COVID-19 pneumonia with high fever (> 38°C) and respiratory symptoms dyspnea and cough.	317	Multiple RT-PCR testing and clinical observation and follow up.	Yes	Yes
Prokop et al. (12)	Prospective collection in emergency department with symptomatic patients	Adult patients with a chest CT scan upon suspicion of COVID-19 pneumonia with high fever (>38°C) and respiratory symptoms dyspnea and cough in that were	105	RT-PCR testing and clinical observation and follow up.	No	No
		followed and in whom RT-PCR was performed				
Schulze-Hagen et al. (49)	Prospective collection in emergency department with symptomatic patients	Adult patients with a chest CT scan upon suspicion of COVID-19 pneumonia with high fever (>38°C) and respiratory symptoms dyspnea and cough in that were followed and in whom RT-PCR was performed	191	RT-PCR testing and clinical observation and follow up.	Yes	Yes
Song et al. (43)	Retrospective analysis in the Emergency department with symptomatic patients	Patients with respiratory symptoms but no significant improvement in conventional anti-infective treatment; clinically suspected to have COVID-19 due to contact history with COVID-19 patients within 14 days before symptom onset or due to clustering onset; or with pending invasive operation in need of routine inspection to exclude COVID-19. Exclusion criteria: the first RT-PCR tested > 3 days before or after CT scan; or incomplete baseline characteristics and laboratory findings.	211	RT-PCR, repeated if initially negative (although this standard could not be applied to ∼34% of initially negative patients that were thus considered negative)	Yes	Yes
Steuwe et al. (44)	Prospective collection in emergency department and hospital setting	Adult patients with a chest CT scan upon suspicion of COVID-19 pneumonia with high fever (>38°C) and respiratory symptoms dyspnea and cough.	105	Repeated RT-PCR, hospitalized patients with two negative RT-PCR test results, a third RT-PCR test was performed from bronchial lavage specimens + daily RT-PCR if CT examination showed typical COVID-19 findings.	Yes	Yes
Wen et al. (45)	Retrospective analysis in the Emergency department with symptomatic patients	Patients with fever > 38.3°C or cough of onset within the last 10 days that required hospitalization. Exclusion criteria: fever for more than 14 days without symptoms and signs for acute respiratory infection or exposure history within 14 days.	103	Multiple sequential PCR tests and observation	Yes	Yes
Xie et al. (46)	Prospective collection in emergency department and hospital setting	Adult patients with a chest CT scan upon suspicion of COVID-19 pneumonia with high fever (>38°C) and respiratory symptoms dyspnea and cough.	19	Multiple RT-PCR testing and clinical observation and follow up.	Yes	Yes
Zhu et al. (47)	Prospective collection in emergency department with symptomatic patients	Adult patients with a chest CT scan upon suspicion of COVID-19 pneumonia with high fever (>38°C) and respiratory symptoms dyspnea and cough in that were followed and in whom RT-PCR was performed. Exclusion criteria; transfer from another hospital or previous visit to the study hospital or previous diagnosis of COVID−19.	116	RT-PCR and if initially negative repeated after 24 h.	Yes	Yes

### Risk of Bias in Included Studies

All included studies were assessed for risk of bias. Tables 2, 3 display the results of the risk of bias assessment. For systematic reviews, the formulation of a specific review question, the search strategies and resources to ensure identification of all relevant studies, methods to minimize errors in data extraction, and methods to combine studies’ results were adequate in most reviews. Nonetheless, all included systematic reviews were found to have at least two items that suggested risk of bias. A frequent issue was the inclusion of primary studies that used single RT-PCR testing as reference standard for all patients, and/or inclusion of studies that only considered patients diagnosed with COVID-19 (hence impairing assessment of false positive, true negative rates, and meta-analyses; not a DTA design). A flawed reference standard, as is the case of single RT-PCR due to high false negative rate, leads to severely biased estimates of sensitivity and specificity, as discussed in the methods section. To include such reports in metanalysis was considered inadequate. Thus, results from systematic reviews that included DTA studies that used single PCR testing as a reference standard were not considered for synthesis; neither were those results from reviews that included studies of only COVID-19-confirmed patients. Four reviews used appropriate selection criteria based on an appropriate reference standard and inclusion of patients independent of that reference standard result (22, 24, 30). One lacked a risk of bias assessment for the included studies and only two specified that this process was performed in duplicate (22, 24, 30). One used a single database as a resource to identify studies and was conducted early in the pandemic, leading to missing studies that were published later (22). These findings pointed to the need to conduct a new metanalysis to overcome those limitations.

**TABLE 2:** Risk of bias assessment for included systematic reviews.

Citation	(1) Is the review question clearly and explicitly stated?	(2) Were the inclusion criteria appropriate for the review question?	(3) Was the search strategy appropriate?	(4) Were the sources and resources used to search for studies adequate?	(5) Were the criteria for appraising studies appropriate?	(6) Was critical appraisal conducted by two or more reviewers independently?	(7) Were there methods to minimize errors in data extraction?	(8) Were the methods used to combine studies appropriate?	(9) Was the likelihood of publication bias assessed?	(10) Were recommendations for policy and/or practice supported by the reported data?	(11) Were the specific directives for new research appropriate?
Adams et al. (22)	Y	Y	Y	N	Y	Y	Y	Y	N	N/A	Y
Böger et al. (5)	Y	N	U	Y	Y	Y	Y	Y	N	Y	N
Huang et al. (24)	Y	Y	Y	Y	N	N/A	Y	Y	N	N/A	U
Islam et al. (31)	Y	N	Y	Y	Y	Y	Y	Y	N	Y	Y
Li et al. (25)	Y	N	Y	Y	Y	N	Y	Y	Y	N	Y
Kim et al. (27)	Y	N	Y	Y	Y	U	Y	Y	Y	Y	U
Lv et al. (28)	Y	N	Y	Y	N	Y	Y	Y	N	Y	Y
Xu et al. (30)	Y	Y	U	Y	Y	Y	Y	Y	N	Y	N/A
Shao et al. (29)	Y	N	N	Y	Y	U	U	N/A	N	Y	Y
Mair et al. (23)	Y	Y	Y	Y	Y	U	Y	Y	N	Y	Y
Khatami et al. (26)	Y	N	Y	Y	N	N/A	Y	Y	Y	N/A	N/A
											

**TABLE 3:** Risk of bias assessment for included diagnostic test accuracy studies.

Study	(1) Was a consecutive or random sample of patients enrolled?	(2) Was a case-control design avoided?	(3) Did the study avoid inappropriate exclusions?	(4) Were the index test results interpreted without knowledge of the results of the reference standard?	(5) If a threshold was used, was it pre-specified?	(6) Is the reference standard likely to correctly classify the target condition?	(7) Were the reference standard results interpreted without knowledge of the results of the index test?	(8) Was there an appropriate interval between index test and reference standard?	(9) Did all patients receive the same reference standard?	(10) Were all patients included in the analysis?
Aslan et al. (32)	Y	Y	Y	U	N/A	Y	Y	Y	N	Y
Caruso et al. (34)	Y	Y	Y	U		Y	Y	Y	Y	Y
Debray et al. (35)	Y	Y	Y	Y		SC	Y	Y	N	Y
Gezer et al. (36)	Y	Y	Y	Y		Y	U	Y	Y	Y
Gietema et al. (37)	Y	Y	Y	Y		Y	Y	Y	N	Y
He et al. (48)	Y	Y	Y	Y		Y	Y	Y	N	Y
Herpe et al. (39)	Y	Y	Y	Y		Y	Y	U	Y	Y
Krdzalic et al. (41)	Y	Y	Y	Y		Y	Y	Y	N	Y
Patel et al. (42)	Y	Y	Y	Y		SC	Y	Y	N	Y
Schulze-Hagen et al. (49)	Y	Y	Y	Y		Y	Y	Y	Y	Y
Song et al. (43)	Y	Y	Y	Y		SC	Y	Y	N	Y
Steuwe et al. (44)	Y	Y	Y	Y		Y	Y	Y	N	Y
Wen et al. (45)	Y	Y	Y	Y		Y	Y	Y	Y	Y
Xie et al. (46)	Y	Y	Y	Y		Y	Y	Y	Y	Y
Zhu et al. (47)	Y	Y	Y	Y		Y	Y	Y	N	Y
										

Regarding primary DTA studies, assessment of risk of bias is presented only for studies considered for metanalysis and is displayed in Table 3. All studies used an adequate reference standard (see methods). Nonetheless, some studies performed further testing and follow up only in patients with initial negative results; while patients that had symptoms and a positive PCR test were considered positive. This is due to the inherent properties of RT-PCR testing (low sensitivity and high specificity), which confer confidence in positive results but skepticism in negative results. This reference standard was also considered adequate. Two studies report being unable to repeat PCR testing for initially negative patients in 1/3 of their study sample. These studies are marked as “with some concerns” (SC) regarding risk of bias.

### Metanalysis and Summary Receiver Operator Characteristics Estimation

**FIGURE 2:**
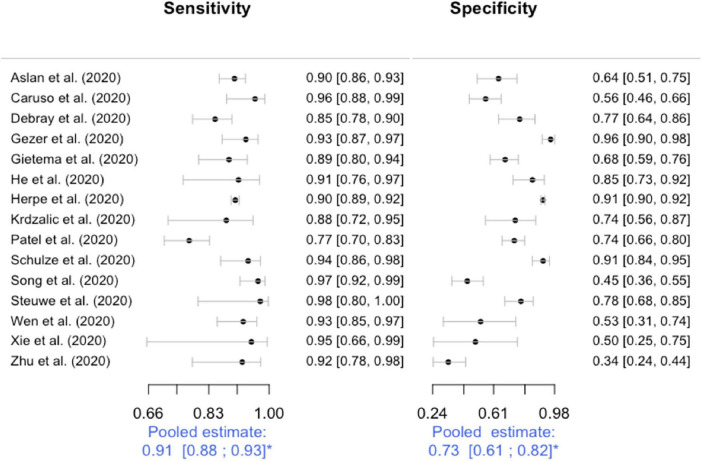
Forest plots for sensitivity and specificity. Studies are sorted alphabetically. *Estimated pooled sensitivity and specificity with their 95% confidence intervals from bivariate methods using a random effects model. As no statistical method is currently available for heterogeneity assessment in diagnostic metanalysis with bivariate methods, heterogeneity was assessed qualitatively and considered low for sensitivity and high for specificity. Although high, heterogeneity for specificity estimates was explained by differences between studies and thus considered not serious (see quality of evidence in the results section). Sensitivity analysis excluding studies with concerns regarding flawed reference standard did not change displayed estimates.

**FIGURE 3:**
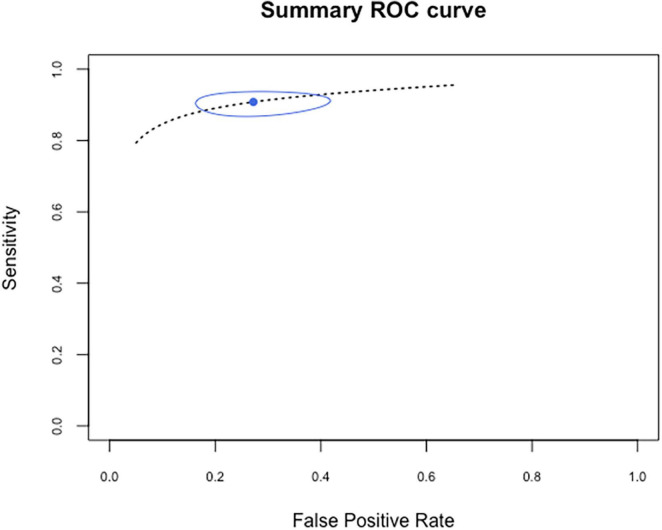
Summary ROC curve. Estimation of diagnostic accuracy of chest CT for detection of COVID-19: high sensitivity with narrow range of variability and modest specificity (inverse of false positive rate) with a wider range of variability; attributable to variable local COVID-19 incidence and differences in reference standards applied.

Of the 19 primary DTA studies 15 were meta-analyzed. Four studies that reported CT findings according to a radiological scale without positive or negative result (index test) are summarized but were not considered in metanalysis (12, 33, 38, 40). Figure 2 displays forest plot of sensitivities and specificities reported by the primary studies; pooled estimates are derived from bivariate metanalysis. Sensitivity of chest CT for COVID-19 was estimated at 0.91, 95%CI = (0.88–0.93). Specificity was estimated at 0.73, 95%CI = (0.61; 0.82). Higher heterogeneity is visually evident for specificity, while sensitivities are more homogeneous. Figure 3 displays the Summary ROC (SROC) curve that demonstrates such variability within a small range of values for sensitivity estimates and within a wider range for specificity estimates. No correlation was found between sensitivities and specificities (ρ = 0.22, IC95% [–0.33; 0.66]). Diagnostic odds ratio was estimated at: DOR = 27.5, 95%CI (14.7; 48.5).

### Likelihood Ratios and Practical Interpretation of Results

The calculated likelihood ratios derived from pooled estimates of sensitivity and specificity were: LR+ = 3.44 (sensitivity/[1-specificity]) and LR– = 0.13 ([1-sensitivity]/specificity). A practical interpretation of this results can be made using the estimated change in pre-test probability according to calculated LRs provided by McGee (50). The presence of a compatible or highly suspicious chest CT (“positive”) increases pre-test probability by 20–25%, while an unlikely/incompatible (negative) chest CT reduces the pre-test probability of COVID-19 by ∼45%.

### Quality of the Evidence

Evidence assessment to estimate diagnostic accuracy of chest CT for COVID-19 was undertaken separately for sensitivity and specificity, as recommended. Both assessments considered 15 studies comprising 4824 patients. Evidence for sensitivity estimates was graded as “Moderate” due to indirectness of evidence, and for specificity estimates it was graded as “Low” due to imprecision (evidenced by a wide 95% confidence interval) and indirectness. The summary of findings (SoF) table (Table 4) displays the judgments made for each aspect of quality assessment and their corresponding explanations. Effect is presented for pre-test probabilities of 1 and 10%.

**TABLE 4:** Summary of findings.

Question: Should Chest CT be used to screen for COVID-19 in patients that require emergency surgery due to trauma?										
	Sensitivity 0.91 (95% CI: 0.88 to 0.93) Specificity 0.73 (95% CI: 0.61 to 0.82)			Prevalences 1% 10%						

Outcome	No of studies (No of patients)	Study design	Factors that may decrease certainty of evidence					Effect per 1,000 patients tested		Test accuracy CoE
					
			Risk of bias	Indirectness	Inconsistency	Imprecision	Publication bias	Pre-test probability of 1%	Pre-test probability of 10%	
**True positives** (patients with COVID-19)	15 studies 4824 patients	Cross-sectional (cohort type accuracy study)	Not serious	Serious ^a^	Not serious	Not serious	None	9 (9–9)	91 (88–93)	⊕⊕⊕○ MODERATE
**False negatives** (patients incorrectly classified as not having COVID-19)								1 (1–1)	9 (7–12)	
**True negatives** (patients without COVID-19)	15 studies 4824 patients	Cross-sectional (cohort type accuracy study)	Not serious	Serious*^a^*	Not serious^b^	Serious^c^	None	723 (604–812)	657 (549–738)	⊕⊕○○ LOW
**False positives** (patients incorrectly classified as having COVID-19)								267 (178–386)	243 (162–351)	

*^a^All DTA studies assessed chest CT for COVID-19 in symptomatic suspected patients. Emergency trauma patients have a much lower pre-test probability of COVID-19. Although accuracy of chest CT is not expected to change, chest trauma may affect image reading, and the different clinical setting constitutes indirect evidence.*

*^b^Although inconsistency of results was observed, it is explained by variable local incidence of COVID-19 cases and by studies that used an imperfect reference standard in up to one third of included patients; both of which affect specificity estimates. As such, we considered that there was not “unexplained heterogeneity,” which is the finding that downgrades quality.*

*^c^Given the issues explained in b, pooled estimates of specificity have a wider confidence interval and were considered imprecise.*

### Additional Findings

Three studies specifically addressed using CT as a method of screening for COVID-19 in an emergency surgery setting. In one study of 28 patients with initial negative RT-PCR results that upon a second test became positive, the mean interval time between negative to positive results was 6.2 days, with some up to 15 days (51). In two cases PCR was negative two times before being positive, with positive chest CT findings identifying lesions on the first day. In countries like India where there is an ongoing surge of COVID-19 patients, shortages in test kit supplies have strained health systems. CT testing can compensate for this, especially in situations where the patient needs emergency surgery. In these cases, the urgency to get the patient to the operating room is incompatible with the time needed to receive test results from PCR. This is important as one study found that there was a higher number of positive PCR in the trauma population than in the general population (52). On the contrary, in low-resource areas in which CT scans are not readily available at many hospitals transferring patients to hospitals with CT availability may be a risk to patients and healthcare workers. Another study of 207 patients admitted for acute surgical emergencies found a negative predictive value of 82.4%, concluding that CT of the thorax has the potential to play an important role in helping surgeons in their decision making (53). However, the authors note that over-reliance on CT with its high false positive rate can lead to overtreatment, overuse of resources and delays in the decision-making process. In the third study with data provided for over 800 patients undergoing both emergency and elective surgical interventions over a 5-day period at all UK hospitals with imaging departments, a high rate of false positives was found, producing a sensitivity of 68.4% for thoracic CT (54). These authors suggest that the diagnostic yield is low and that additional CT examinations expose patients to an unnecessary extra dose of radiation to the patient.

### The CO-RADS Scale

The most widespread method for the diagnosis of COVID-19 using CT imaging appears to be the COVID-19 Reporting and Data System (CO-RADS), introduced by the Radiological Society of the Netherlands (NVvR) and largely based on the recommendations of the Radiological Society of North America (11, 12). The scoring system uses a scale from 0 to 5 to grade the level of suspicion of COVID-19 infection based on pulmonary involvement from very unlikely to very likely. In a study of 105 patients, the NVvR found high performance for predicting COVID-19 with an AUC of 0.91 (CI, 0.85–0.97) (12, 55). A high negative predictive value and low negative likelihood ratio was associated with a CO-RADS ≤ 3 while a high positive likelihood ratio and good positive predictive value was associated with a CO-RADS score ≥ 4. It is important to note that in both studies the prevalence of COVID-19 was high. As cases drop it is likely that the false-positive rate and negative predictive value will increase. Of the reviewed studies, in four that did not report a dichotomous index test (+ or –) and thus could not be meta-analyzed, the CO-RADS scale was used (12, 33, 38, 56). Dichotomization of the scale was used by one additional study that did report + or – results for the index test.

### Interrater Agreement of Chest Computed Tomography for COVID-19

Since diagnostic efficacy is dependent upon the radiologist’s interpretation of the CT scan, the reproducibility of the categorization of CT reports among multiple observers is an essential component when considering appropriate clinical decision making. In a study of 241 COVID-19 suspected patients, eight observers categorized each CT into one of four categories (evocative, compatible for COVID-19 pneumonia, not evocative, and normal) (35). Agreement across the 4 categories was good between all readers (κ value 0.61 95% CI 0.60–0.63) and moderate to good between pairs of readers (0.54–0.75). Among patients considered for hospitalization, CT categorized as evocative is highly predictive of COVID-19, while almost a third of patients with CT categorized as not evocative had a positive RT-PCR. In another study of 34 COVID-19 and 48 non-COVID-19 patients identified by RT-PCR, two radiologists had a good interobserver agreement (κ value 0.69) with 26/34 COVID-19 patients correctly diagnosed at final agreement (48). Since several studies did not examine inter-observer variability of CT findings it is possible that the observed specificity and sensitivity are overestimated.

## Discussion

This review identified, appraised, and summarized the available evidence regarding the diagnostic accuracy of chest CT scan for the diagnosis of COVID-19 in order to inform clinical decisions and recommendations regarding its application in an emergency trauma setting. No studies that assessed chest CT diagnostic accuracy in this specific setting were found. Nonetheless, consideration of evidence from the ED setting during the pandemic was planned in advance given that we judged the diagnosis accuracy largely extrapolatable, as is indicated in our published protocol (8). Some case series reporting the utility of CT in the pre-operative screening on COVID-19 were summarized narratively but were not a source for diagnostic accuracy of the test. We report that chest CT is a highly sensitive tool to detect COVID-19 in suspected patients and would be expected to have a similar sensitivity when applied to a trauma patient, but has lower specificity.

Great variation was present between studies due to differences in design, index test definition and reference standards. The nature of the disease and differences in settings likely affected specificity estimates in the included studies. The local incidence of COVID-19 cases explains variability of specificity estimates among studies. When incidence is low, abnormal chest CT findings suggestive of COVID-19 might be more often caused by other etiologies such as other respiratory viruses. Conversely, in a context of high COVID-19 incidence, abnormal findings due to non-COVID-19 pneumonias constitute a smaller proportion of the studied patient. This leads to a lower false positives rate being recorded for the test, and hence, higher calculated specificity. This relationship is to be considered when applying and interpreting results of chest CT for screening or diagnosing COVID-19.

The value of chest CT appears to be that of an additional screening tool that can easily detect PCR false negatives, which are reportedly frequent. It is a sensitive tool to diagnose COVID-19, and specificity can vary as discussed. Carrier/asymptomatic status, which is believed to also represent contagion risk, is obviously not expected to be detected with chest CT. Thus, a negative chest CT does not exclude SARS-CoV-2 transmission in the incubation phase. However, given the absence of COVID-19 pneumonia, the likelihood of contagion is significantly reduced. In this sense, CT can have increased value if reading is compatible with COVID-19 when either PCR testing is unavailable or results are delayed. Compatible findings on chest CT should prompt additional protective measures in aerosolizing procedures for medical staff and isolating measures for the patient should be considered. As emergency trauma patients typically undergo localized or full body CT scanning, imaging of the lungs and its interpretation by a radiologist is not expected to increase costs significantly and can be implemented as a screening tool in that setting. One important consideration are patients with trauma or polytrauma involving the chest; as lung contusions and hemo- or pneumo-thorax will affect the readability of chest CT for pneumonia.

### Strengths and Limitations

A considerable proportion of the systematic reviews encountered when conducting this synthesis were found to be of low methodological quality and thus with high risk of bias because they included studies without a DTA design where a reference standard and an index test are applied to all patients, studies of only COVID-19 positive cases and/or did not considered the rate of false negatives on single initial RT-PCR. We provide new estimates that overcome these limitations and that should serve as a trustworthy source of information regarding the diagnostic accuracy of chest CT for COVID-19. We hope that the insights into the methodology of assessing diagnostic tests performance and synthesizing this type of evidence will be of value for readers.

Quality of the evidence for diagnostic accuracy of chest CT was moderate for sensitivity estimates and low for specificity estimates, mostly due to indirect evidence and, in the case of specificity, imprecision. Higher quality of evidence requires studies that assess chest CT in the trauma setting. Chest CT is a sensitive test for COVID-19 that can have a role in screening of trauma patients with need for urgent surgical care; it has easy implementation as CT is routinely performed in trauma patients, and could be particularly useful in low-resources settings where supplies are to be used selectively.

## Data Availability Statement

The original contributions presented in the study are included in the article/supplementary material, further inquiries can be directed to the corresponding author/s.

## Author Contributions

All authors listed have made a substantial, direct, and intelectual contribution to the work, and approved it for publication.

## Conflict of Interest

The authors declare that the research was conducted in the absence of any commercial or financial relationships that could be construed as a potential conflict of interest.

## Publisher’s Note

All claims expressed in this article are solely those of the authors and do not necessarily represent those of their affiliated organizations, or those of the publisher, the editors and the reviewers. Any product that may be evaluated in this article, or claim that may be made by its manufacturer, is not guaranteed or endorsed by the publisher.

## Abbreviations

CT: computed tomography
ED: emergency department
PRISMA: preferred reporting items for systematic reviews and meta-analyses
LOVE: living overview of the evidence
DTA: diagnostic test accuracy
WHO: World Health Organization
PCR: polymerase chain reaction
UK: United Kingdom
CO-RADS: COVID-19 reporting and data system
NVvR: radiological society of the Netherlands.
